# Applicability of adipose-derived mesenchymal stem cells in treatment of patients with type 2 diabetes

**DOI:** 10.1186/s13287-019-1362-2

**Published:** 2019-08-28

**Authors:** Yicheng Qi, Jing Ma, Shengxian Li, Wei Liu

**Affiliations:** Division of Endocrinology and Metabolism, Department of Internal Medicine, RenJi Hospital, School of Medicine, Shanghai Jiaotong University, 160# Pujian Road, Pudong, Shanghai, 200127 China

**Keywords:** T2DM, Insulin resistance, β cell, MSC therapy, AD-MSCs

## Abstract

Type 2 diabetes mellitus (T2DM) is mainly characterized by insulin resistance (IR) and impaired insulin secretion. The chronic inflammatory process contributed to IR and could also hamper pancreatic β cell function. However, currently applied treatment cannot reverse β cell damage or alleviate inflammation. Mesenchymal stem cells (MSCs), the cell-based therapy for their self-renewable, differentiation potential, and immunosuppressive properties, have been demonstrated in displaying therapeutic effects in T2DM. Adipose-derived MSCs (AD-MSCs) attracted more attention due to less harvested inconvenience and ethical issues commonly accompany with bone marrow-derived MSCs (BM-MSCs) and fetal annex-derived MSCs. Both AD-MSC therapy studies and mechanism explorations in T2DM animals presented that AD-MSCs could translate to clinical application. However, hyperglycemia, hyperinsulinemia, and metabolic disturbance in T2DM are crucial for impairment of AD-MSC function, which may limit the therapeutical effects of MSCs. This review focuses on the outcomes and the molecular mechanisms of MSC therapies in T2DM which light up the hope of AD-MSCs as an innovative strategy to cure T2DM.

## Background

Diabetes mellitus (DM) is a worldwide epidemic and increases rapidly in recent years. It is estimated that adults with diabetes increased from 108 million in 1980 to 422 million in 2014 in the world [1]. DM affects multiple organ systems and causes variety of vascular and several nonvascular complications, which are the major cause of premature deaths [2]. Type 2 diabetes mellitus (T2DM), which accounts for 85–95% of all cases, is characterized by insulin resistance (IR) in insulin-responsive tissues and impaired insulin secretion from the pancreatic β cell [3]. As the disease course, β cell function is reduced at a rate of 6~8% yearly, playing the key role in the occurrence and development of T2DM [4]. It has been demonstrated that the pathogenesis of T2DM is related to systemic low-grade chronic inflammation. Overnutrition first leads to adipose tissue to undergo hypertrophic enlargement, which results in imbalance of pro-inflammatory cytokines and anti-inflammatory cytokines, promoting immune cell infiltration. Inflammatory factors and chemokines of adipose tissue then are released to the circulation and affect a variety of tissues including the β cells, adipose tissue, liver, and muscle, ultimately leading to the deteriorated insulin-sensitive and beta cell function [3, 5].

Initial treatments of T2DM typically include lifestyle modification and oral hypoglycemic agents. However, many patients gradually have difficulties to achieve ideal glycemic control. These patients eventually need insulin to lower blood glucose levels [6]. T2DM patients often need the medicines for the rest of their lives. Accordingly, the risk of adverse effects should be concerned. Most importantly, these treatments only decrease glycemic levels, but have no effects on diabetes and diabetic complications for irreversible pancreatic β cell impairment [7]. Therefore, it is extremely imminent to explore alternative approaches to cure diabetes and maintain therapeutic effect.

Pioneer strategies for β cell replacement including pancreatic transplantation and islet cell transplantation have been performed for clinical application [8]. With the advance of surgical technique and immunosuppression, 1-year insulin independence rates after pancreas transplantation greatly were improved from 52% in the 1990s to 78% around 2010. Recent 5-year graft function rates according to the International Pancreas Transplant Registry (IPTR) were 55% [9, 10]. Until now, outcomes of islet transplantation were inferior to whole pancreas transplantation. Data from the Collaborative Islet Transplant Registry (CITR) have shown that insulin-free rate was 66%, 55%, and 44% at 1, 2, and 3 years after islet transplantation, respectively, in 2007–2010 [11]. However, both methods are mainly used for T1DM, and the application experience in patients with T2DM is limited. In addition, these procedures suffer many obstacles, including lack of appropriate donors, surgical techniques, complications of life-long immunosuppressive agents, and exhaustion of the transplanted organs and cells.

In recent years, it has been proven that stem cells are characterized by self renewal, differentiation into other cell lines, and immunoregulation [12–14]. The cell-based therapies have offered a new paradigm in the management of T2DM for creating an unlimited source of insulin-producing cells, repairing β cell function, modulating metabolism, and improving immune dysfunction [15–17]. However, there are various types of stem cell with different potency and sources; thus, the therapeutic effects and utility were different. In this article, we focused on exploring the best choice of stem cell for T2DM treatment and insight into the underlying mechanism.

## Stem cells

Stem cells are biological cells with clonogenic potential that can self-renew and differentiate into multiple types of cells. They are responsible for the regeneration and development of organs and tissues. There are three types of stem cells, including embryonic stem cells (ESCs), adult stem cells (ASCs), and induced pluripotent stem cells (iPSCs). According to differentiation potency, stem cells can be divided into totipotent, pluripotent, multipotent, oligopotent, and unipotent [18]. Human pluripotent stem cells include ESCs and iPSCs. ESCs derived from blastocyst are pluripotent stem cells that can differentiate into almost all kinds of cells; thus, these cells are good option for tissue generation [19]. ESCs have ethical and legal issues as their origin is from human embryos. Moreover, they bear the clinical risks of teratomas and autoimmune response in vivo [20]. iPSCs are a further source of pluripotent stem cells [21, 22]. iPSCs are created by ectopic expression of pluripotency factors such as OCT4, NANOG, SOX2, c-Myc, and KLF44 and more forced by viral vector or non-viral reprogramming factors, thus might with genomic instability [23, 24]. Besides, iPSCs can induce T cell-dependent immune response. Therefore, the immunogenicity should be evaluated before autologous iPSC transplantation [25]. ASCs obtained from practically all organs and tissues are less potent to differentiate into numerous cell types than the other two stem cell types [26–28]. ASCs are generally multipotent, though some of them express pluripotent markers, whereas some ASCs are even oligopotent or unipotent [29, 30]. As shown in Table 1, ASCs can avoid the mutational and potential side effects correlated with iPSCs. Meanwhile, the immunogenicity of ASCs was the lowest among the three stem cell types [31]. Moreover, ASC application can overcome the ethical and legal issues as they can be isolated from an autologous form. These show ASCs were the most advantageous choice for clinical applications.

**Table 1:** The merit and demerit of three stem cell types

	Differentiation potency	Mutation effects	Ethical and legal issues	Immunogenicity
ESCs	Pluripotent	None	Yes	Elicits autoimmune response
ASCs	Multipotent to unipotent	None	None	Low
iPSCs	Pluripotent	Yes	None	Immune response

Mesenchymal stem cells (MSCs) of mesodermal origin, also referred to as “mesenchymal stromal cells,” are ASCs with multidirectional differentiation potential [32]. MSCs could be originated from almost all types of tissue, including the bone marrow, fetal annexes, adipose tissue, dental tissues, skeletal muscle tissue, liver tissue, lung tissue, and menstrual blood [33–38]. MSCs are characterized as (1) plastic-adherent and spindle-shape, (2) expression of antigen markers (CD73+CD90+CD105+CD45−CD34−CD14−CD79−HLA-DR−) on their surface, (3) differentiation potential into adipocytes, osteocytes, and chondrocytes [39]. Despite multipotent stem cells are considered to differentiate to only one germ layer, it has been demonstrated that MSCs can differentiate to non-mesodermal cells in vitro, including pancreatic islet-like cells, neuron-like cells, and hepatocytes [40–42]. MSCs now attract significant attention for their efficacy and safety in cell-based therapy.

## Clinical applications of MSCs in T2DM

The ethical concerns of ESCs and carcinogenic risks of iPSCs limit their application in clinical. MSCs avoid these disadvantages and are currently the most studied cells in T2DM therapy. Recently, some small sample of phase I/II clinical trials using MSCs of different sources have performed for the treatment of T2DM (Table 2). Bone marrow stem cells are the firstly discovered and well-studied stem cells. Early focus are more accessible bone marrow-derived mononuclear cells (BM-MNCs) which contain various stem cell types including hematopoietic stem cells, MSCs, and endothelial progenitor cells. The first study in T2DM was a prospective phase I study with combined therapy of intra-pancreatic BM-MNC infusion and hyperbaric oxygen treatment (HOT) [43]. This trial showed that the combination therapy gradually improved metabolic variables and reduced insulin requirements in patients with T2DM over 1-year follow-up. However, a randomized, open-label, controlled clinical trial revealed that HOT does not synergize with BM-MNC infusion [44]. Several studies indeed have demonstrated the efficacy of stem cell transplantation alone in T2DM. Bhansali et al. similarly found that insulin requirement was reduced by ≥ 50% in 70% patients. Thirty percent of the patients were able to discontinue insulin use completely, accompanied by weight loss and β cell function improvement after intra-pancreatic BM-MNC transplantation [45]. Bhansali et al. thereafter conducted a randomized placebo-controlled study and improved the stem cell therapy method by twice treatment. Patients in the intervention group received BM-MNCs through intra-pancreatic injection, and a second injection of peripheral MNCs was administered through the antecubital vein after 12 weeks. 82% patients in the intervention group achieved reduction in insulin requirement by ≥ 50%, whereas none of the patients in the conventional group did [48]. Recently, Bhansali et al. compared the efficacy and safety of BM-MSC and BM-MNC transplantation in T2DM. It explored that both groups result in sustained insulin requirement reduction in almost 67% patients which due to MSCs improve insulin sensitivity accompanied with increased IRS-1 gene expression, whereas MNCs increase second phase C-peptide response during hyperglycemic clamp [49]. To observe the long-term effect of MSC treatment, Hu et al. studied with a larger sample and found that HbA1c level gradually decreased and reached the lowest level at the end of the first year (baseline 7.95 ± 0.64%, 12 months 6.70 ± 0.70%) after MNC therapy. However, there were slight fluctuations over the following 2 years. Meanwhile, C-peptide was improved 1 year after implantation, and a decrease trend of C-peptide was showed in the next 2 years [47]. Wang et al. found the similar result [46]. It was also notable that BM-MNCs/MSCs were extracted by invading femur or iliac crest, and the procedure was painful with low quantities and increased risk of infection [56].

**Table 2:** Characteristics of the MSC-based therapies in T2DM

Stem cell type	With control study	Number of patients	Sex male/female	Mean age (years)	Mean history of disease (years)	Mean dose of injected cells	Mode of injection	Mean follow-up period	Glycometabolic control	β cell function	Insulin sensitivity	Immunological recovery	Complications	Author
BM-MNCs	–	25	17/8	55.8	13.2	–	Intra-pancreatic	12 months	4/15 insulin-free, 13/15 reduced insulin requirements ≥ 50%, HbA1C decreased 29.5%	C-Pep improved	–	–	No adverse effects	Estrada et al. [43]
BM-MNCs	Yes	Ctrl 20 MNCs 20	11/9 12/8	54.9 56.4	9.5 9.8	MNC 4.0 × 10^9^	Intra-pancreatic	12 months	Insulin dose decreased 30%, HbA1C decreased 13% in the treated group	FCP improved	–	–	Transient abdominal pain Punctual hemorrhage	Wu et al. [44]
BM-MNCs	–	10	8/2	57.5	14.6	MNC 3.5 × 10^8^ (CD34+ 3.1 × 10^6^)	Intra-pancreatic	6 months	3/10 insulin-free, 7/10 reduced insulin requirements ≥ 50%, HbA1C decreased 13.1%	FCP, glucagon-stimulated C-Pep improved, HOMA-β increased	HOMA-IR no change	–	Hematoma Hemoglobin Respiratory infection	Bhansali et al. [45]
BM-MNCs	–	31	–	–	–	MNC 3.76 × 10^9^	Intra-pancreatic	24 months	7/26 reduced insulin requirements, HbA1C decreased 18.4%	C-Pep improved	–	–	–	Wang et al. [46]
BM-MNCs	Yes	Ctrl 62 MNCs 56	36/18 38/18	50.2 50.4	7.3 8.6	MNC 2.8 × 10^9^	Intra-pancreatic	33 months	18/56 insulin-free, 37/56 reduced insulin requirements ≥ 50%, HbA1C decreased 13.1% in treated group; Insulin dose increased gradually in control	FCP, PCP improved in treated group; decreased in control	–	–	No adverse effects	Hu et al. [47]
BM-MNCs +P-MNCs	Yes	Ctrl 10 MNCs 11	7/3 9/2	54 51	20 12	MNC 2.9 × 10^8^ (CD34+ 3.2 × 10^6^) P-MNC 4.9 × 10^8^ (CD34+ 5.8 × 10^6^)	Twice treatment Intra-pancreatic IV	12 months	9/11 reduced insulin requirements ≥ 50%, 10/11 maintain HbA1c < 7% in treated group	Glucagon-stimulated C-Pep improved in treated group, HOMA-β no difference	HOMA-IR lower in treated group	–	Punctual related effects	Bhansali et al. [48]
BM-MNCs/MSCs	Yes	Ctrl 10 MNCs 10 MSCs 10	6/4 7/3 8/2	53.5 44.5 50.5	14 13 15	MNC 1.1 × 10^9^ (CD34+ 1.8 × 10^7^) MSC 8.35 × 10^7^	Intra-pancreatic	12 months	6/10 reduced insulin requirements ≥ 50%, maintain HbA1c < 7% in both treated groups; None improved in the control group	Glucagon-stimulated C-Pep improved in the MNC group	ISI improved in MSCs group	–	Punctual hemorrhage Hypoglycemic	Bhansali et al. [49]
WJ-MSCs	–	22	15/7	52.9	8.7	1 × 10^6^/kg	Twice treatment IV Intra-pancreatic	12 months	7/17 insulin-free, 12/17 reduced insulin requirements ≥ 50% HbA1C decreased 15%	FCP improved; HOMA-β increased	–	T lymphocytes decreased, IL-6 and IL-1β reduced	Punctual hemorrhage Fever C-pep temporary decreased	Liu et al. [50]
WJ-MSCs	–	6	6/0	40.5	3.6	0.88 × 10^6^/kg 0.87 × 10^6^/kg	Twice treatment IV	24 months	3/6 insulin-free,	FCP, PCP improved	–	–	No adverse effects	Guan et al. [51]
WJ-MSCs	Yes	Ctrl 30 MSC 31	16/14 17/14	53.2 52.4	8.3 8.93	1.0 × 10^6^/kg	Twice treatment IV	36 months	10/31 insulin-free, 18/31 reduced insulin requirements ≥ 50%, HbA1C decreased 25.8% in treated group; Insulin dose increased gradually in control	FCP improved; HOMA-β increased	HOMA-IR decreased trend	–	Hypoglycemia	Hu et al. [52]
UC-MSCs	–	18	–	–	–	1 × 10^6^/kg	Thrice treatment IV	6 months	Insulin requirements without reduced	C-Pep without improved	–	Tregs increased trend	Fever	Kong et al. [53]
PD-MSCs	–	10	7/3	66	11	1.35 × 10^6^/kg	Thrice treatment IV	6 months	4/10 reduced insulin requirements ≥ 50%, HbA1C decreased 31.6%	C-Pep improved	–	–	No adverse effects	Jiang et al. [54]
CB-SCs	–	36	21/15	52	14	Stem cell educator therapy		14 months	Insulin dose, oral medications, HbA1C decreased	FCP improved; HOMA-β increased	HOMA-IR reduced	TGF-β1, CTLA-4 increased; IL-17, IL-12,IL-4, IL-5,CD86 + CD14+ monocytes decreased; increased	Punctual-related effects	Zhao et al. [55]

*Abbreviations*: *MNCs* mononuclear stem cells, *MSCs* mesenchymal stem cells, *BM* bone marrow, *P* peripheral, *WJ* Wharton’s jelly, *UC* umbilical cord, *PD* placenta-derived, *CB-SC* cord blood-derived multipotent stem cells, *Ctrl* control, *IV* intravenous, *FCP* fasting c-peptide, *PCP* postprandial c-peptide, *C-Pep* c-peptide, *HOMA-β* homeostatic model assessment of beta cell function, *HOMA-IR* homeostasis model assessment of insulin resistance, *ISI* insulin sensitivity index

Fetal annex-derived MSCs have higher similarity to ESCs, but greater differentiation potential than the other common MSC types. Several studies have observed the efficacy and safety of fetal annex-derived MSCs. T2DM patients received Wharton’s jelly-derived MSC (WJ-MSC) transplantation via one intravenous injection and one intra-pancreatic endovascular injection (catheterization). This therapy not only improved metabolic control, and β cell function, but also reduced inflammatory cytokines and T cell counts [50]. Guan et al. showed that twice intravenous injection of WJ-MSCs improved the diabetic status by increasing C-peptide and decreasing HbA1c levels [51]. Interestingly, regulatory T (Treg) cell number showed there was an increased trend after umbilical cord–derived MSC (UC-MSC) infusion, together with slightly reduced insulin requirements [53]. Similar to the long-term effect of BM-MSCs, WJ-MSCs would cause slight fluctuation in the late period of follow-up. HbA1c gradually decreased in the patients with WJ-MSC infusion, and the lowest level was at the sixth month of follow-up, after which HbA1c remained stable for 18 months, subsequently exhibited slight fluctuations over the remaining follow-up period. Fasting serum C-peptide reached the peak levels at 3 months, followed by a slight decrease after 18 months. WJ-MSC infusion also simultaneously reduced the incidence of diabetic complications, including diabetic retinopathy, neuropathy, and nephropathy [52]. Jiang et al. even investigated placenta-derived MSC (PD-MSC) therapy in long-standing T2DM. After thrice intravenous infusions of PD-MSCs, 40% responders attained insulin dose reduction of ≥ 50%; meanwhile, the renal function and cardiac function were improved [54]. In addition, Stem Cell Educator therapy, an innovative technology, was designed to control or reverse immune dysfunctions in T2DM. It consists of a closed-loop system that circulates a patient’s blood through a blood cell separator, briefly co-cultures the patient’s lymphocytes with adherent cord blood-derived multipotent stem cells (CB-SCs) in vitro, and returns the educated lymphocytes (but not the CB-SCs) to the patient’s circulation. Zhao et al. found that Stem Cell Educator therapy achieved metabolic improvement and inflammation reduction by modulating monocytes and balancing Th1/Th2/Th3 cytokine production [55]. However, fetal annex-derived MSCs were extracted just after birth, which has to face the potential risk of opposed to allogeneic stem cells and ethical problems.

After BM-MSCs and fetal annex-derived MSCs, adipose-derived MSCs (AD-MSCs) become the alternative choice for clinical cell therapy because of easy accessibility, abundant sources, subcutaneous location, and longer incubation time [57, 58]. In comparison with BM-MSCs, AD-MSCs are harvested with less pain, have senescence later, are superior to maintaining proliferating ability and differentiation potential, and have an approximate threefold increase in immunosuppressive activity [59–64]. Microarray analysis revealed that 1% of genes were differentially expressed between AD-MSCs and BM-MSC [65]. Further comparison with fetal annex-derived MSCs, AD-MSCs have less ethical problems. Therefore, AD-MSCs may be a better candidate for clinical application in theory. There were few clinical studies or ongoing trials about AD-MSC therapy in T2DM. The only Chinese study found that FPG, 2 h-PBG, and HbA1c in the AD-MSCs group decreased more than those in conventional treatment. C-peptide was also improved in AD-MSC therapy [66]. Besides, animal experiments have demonstrated that AD-MSC infusion could improve hyperglycemia through recovery of islet β cells, reduction of inflammation, and improvement of insulin sensitivity [15, 16]. It is also worth noting that some mechanism exploration provide potential of AD-MSC clinical application [67–70].

## The mechanisms of AD-MSCs in T2DM

**Fig. 1:**
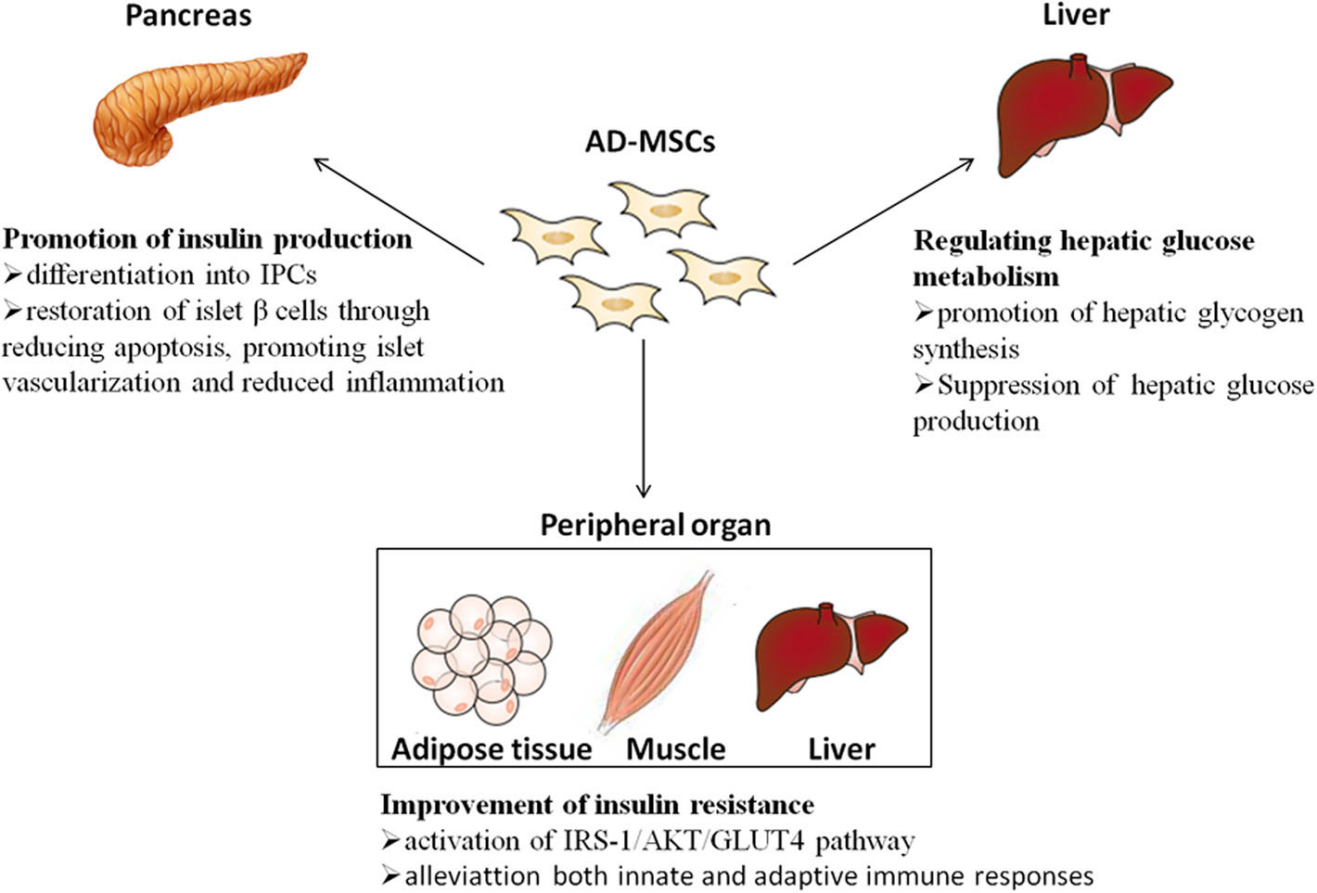
The underlying mechanisms of AD-MSC effect on T2DM. AD-MSCs improve T2DM through promotion pancreatic islet β cell function, amelioration of insulin resistance of peripheral tissue, and regulation hepatic glucose metabolism

In the last decades, we continue to have faith from the MSC therapeutic approaches. The unique property of MSCs is regeneration including regenerating cells, tissues, and organs. Immunomodulatory and immunosuppressive effects is another important property of MSCs, which make the MSC-based therapy useful in inflammatory and autoimmune disorders [71–73]. The potential mechanisms of AD-MSC application in T2DM are summarized in Fig. 1.

### Promotion of insulin production

In T2DM, MSCs from various sources have the potential to differentiate into insulin-producing cells (IPCs). The effect can be ameliorated by hyperglycemia. The differentiation program was controlled by activating key transcription factors such as Pdx-1, Pax4, Pax6, Ngn-3, NeuroD1, and Isl-1 [74]. Some of the transcription factors including Isl-1 and Pax-6 were also expressed in AD-MSCs, which indicated AD-MSCs are capable to differentiate into the IPCs to cure diabetes [67]. Chandra et al. first induced AD-MSCs into IPCs using murine epididymal (mE)-ASCs of Swiss albino mice. After 10 days of culture with differentiation cocktails, AD-MSCs progressively differentiated into cells expressing insulin in a glucose-dependent manner. These IPCs transplanted into experimental diabetic mice bring about normoglycemia within 2 weeks [68]. The same phenomenon was observed by Nam et al. After differentiating human eyelid AD-MSCs into IPCs in vitro, cells were transplanted into a T2DM mouse model. Compared to undifferentiated the AD-MSCs and sham group, IPCs group mice showed better therapeutic effect in improving glucose level by increasing circulating insulin level and ameliorating metabolic parameters including IL-6 [17]. However, only a small portion of MSCs homing to the pancreas after MSC therapy and few of the cells can express insulin, which may not be sufficient to explain the regenerated β cells [75]. AD-MSCs also promote restoration of islet function and increase of islet β cells. The AD-MSCs repair islet cells by reducing the rate of apoptosis through decreasing caspase-3 activity. Meanwhile, the release of paracrine angiogenic factors such as VEGF, IGF-1, HGF, and Vwf can promote islet vascularization and then participate in the cell regeneration [16]. Wang et al. demonstrated pancreatic β cell mass increased after AD-MSC infusion which is associated with less inflammation in the pancreas by reducing TNF-α expression [15].

### Improvement of insulin resistance

In addition to pancreatic islet β cell dysfunction, insulin resistance also plays a crucial role in the process of T2DM. Hu et al. also found that AD-MSC transplantation alleviated hyperglycemia and insulin resistance in a high-fat diet/STZ-induced T2DM rat model by restoring GLUT4 and INSR on the cell membrane in skeletal muscle, liver, and adipose tissues with increased phosphorylation of IRS-1 [16]. Similar with MSCs, the secretome from conditioned media (CM) of stem cells also has the potential to treat various disorders. CM of AD-MSCs has been reported to restore insulin level and stimulate glucose uptake via improving insulin sensitive. The effects are due to obvious enhancement of GLUT4 gene and p-Akt protein, significant reduction of IL6 and PAI1 gene in RI cell models, accumulation reduction of intramuscular triglyceride in C2C12 cells, and adipogenesis inhibition in 3T3L1 cells after CM treatment [69].

Insulin resistance is related to obesity-associated chronic inflammation. Inflammatory cytokines may inhibit IRS and PI3K subunit wire/threonine residues phosphorylation in insulin signaling pathway, leading to signal transmission blocked and IR occurrence [76]. MSCs have been shown to modulate both innate and adaptive immune responses [12, 77]. MSCs can promote the polarization of macrophages from a 0pro-inflammatory phenotype to an anti-inflammatory phenotype through the production of immunosuppressive molecules and metabolites. In T2DM rats, UC-MSCs can alleviate insulin resistance in part via production of IL-6 that elicits M2 polarization [78, 79]. Moreover, MSCs can inhibit the infiltration of macrophages, monocytes, and neutrophils into sites of inflammation in a TSG6-dependent manner [80, 81]. MSCs also have the direct or indirect effect on adaptive immune response by regulating T cell proliferation, survival, and differentiation which is licensed by inflammatory cytokines [82, 83]. Interestingly, the immunoregulation on T cells by MSCs is a dual direction dependent on the inflammatory balance of the MSCs reside microenvironment [84]. MSCs suppress inflammatory response in the presence of intense pro-inflammation, while weaken the MSC suppression with the presence of anti-inflammatory molecules. After AD-MSC infusion, it has been reported that TNF-α, IL-6, and IL-1β significantly decreased in T2DM rats [16]. The impact of AD-MSC infusion on liver steatosis also has been measured. Injection of AD-MSCs reduced liver weight and steatosis by inhibiting pro-inflammatory genes including IL-6, TNF-α, and F4/80 expression (represented macrophage infiltration) and alleviating insulin resistance might via increasing IRS expression [15]. Therefore, the immune regulation effect of MSCs is one of the strategies for AD-MSC therapy.

### Regulating hepatic glucose metabolism

Xie et al. indicated that AD-MSC infusion rapidly alleviated hyperglycemia within 24 h in T2DM rats. This acute effect of AD-MSCs in restoring glucose homeostasis in T2DM could not be completely explained by improving β cell function and insulin resistance. The liver plays important roles in maintaining normal glucose level via regulation of glycogen metabolism and gluconeogenesis. Xie et al. further demonstrated that the glucose metabolism-related enzymes levels are increased in the livers of T2DM rats within 24 h after AD-MSC infusion. It depended on AMPK signaling pathway, thus rapidly reduced glucose levels by promoting hepatic glycogen synthesis and suppressing hepatic glucose production. These data remind us the rapid effect of AD-MSCs in glucose homeostasis [70].

## The effects of T2DM on AD-MSCs

**Fig. 2:**
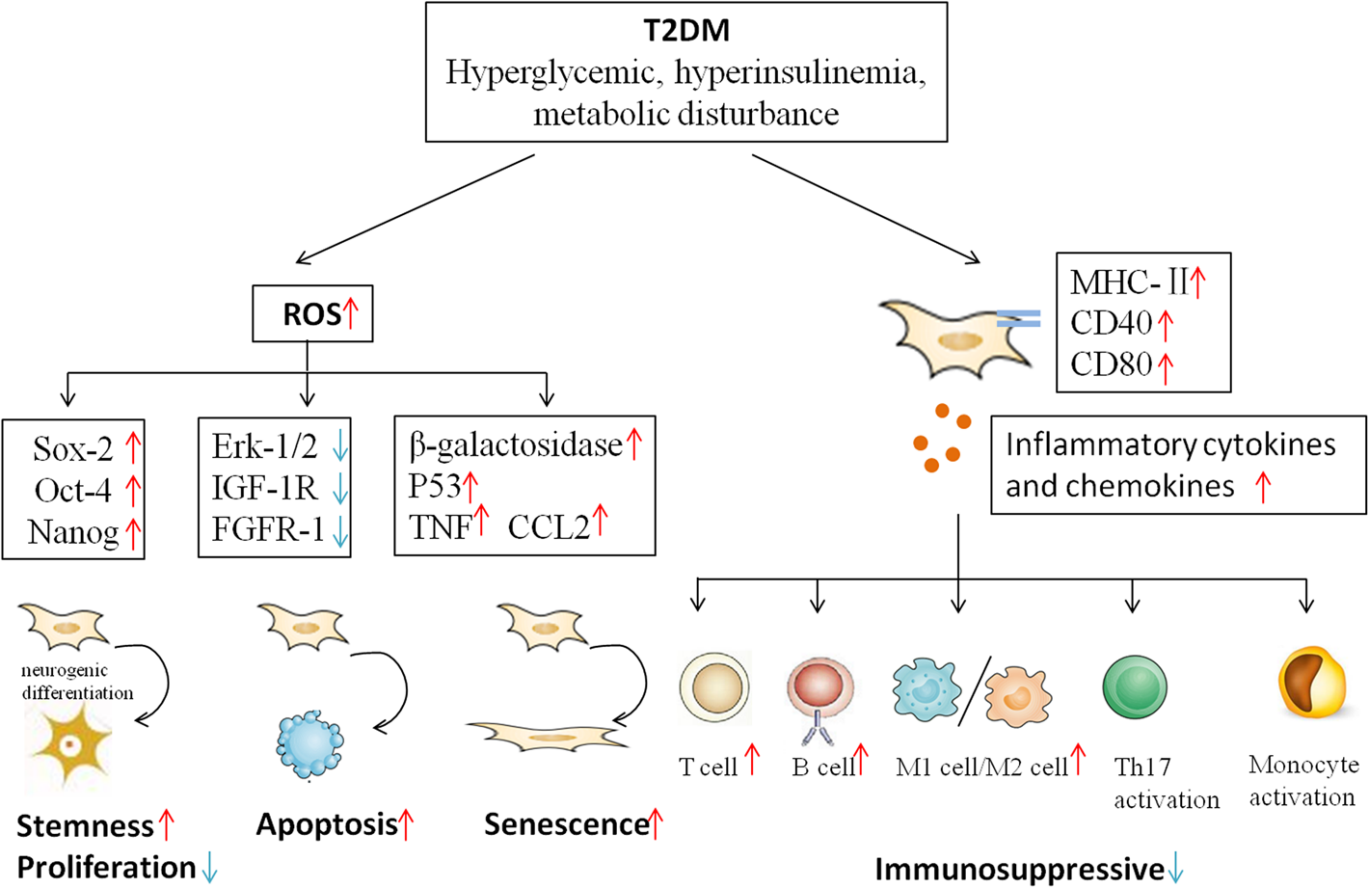
The effects of T2DM on AD-MSCs. T2DM-related metabolic dysfunction impair AD-MSC functionalities including undifferentiated multipotent potential, proliferation, apoptosis, senescence, and immunomodulation

Hyperglycemic and metabolic disturbance microenvironment plays a critical role in damaging several organs and cell types including MSCs, which is an obstacle of autologous AD-MSC clinical application. Systems biology approach has identified the alteration of gene expression including MSC-specific markers, stemness markers, stem cell maintenance molecules, and cellular movement molecules in AD-MSCs and subcutaneous adipose tissue (SAT) in a T2DM rat model [85]. The main influences of T2DM on AD-MSCs are summarized in Fig. 2.

### Diabetic state influences ROS system

Reactive oxygen species (ROS) is involved in modulation of cell physiological differentiation, proliferation, and migration, as well as diseases including diabetes, hypertension, and degenerative diseases [86]. Excessive calorie intake led to the accumulation of ROS in the adipose tissue (the original source of AD-MSCs) of mice with type 2 diabetes and promoted senescence-like changes by increasing activity of senescence-associated β-galactosidase, expression of p53, and production of pro-inflammatory cytokines [87]. It has been reported that diabetic AD-MSCs and high glucose-treated control AD-MSCs displayed enhanced stemness by upregulation of pluripotent genes Sox-2, Oct-4, and Nanog through the stimulation of intracellular ROS synthesis, despite decreased proliferation [88]. Not only hyperglycemia, but high insulin level in diabetes can cause the ROS accumulation. Scioli et al. demonstrated that increased AD-MSC apoptosis induced by high insulin level in a dose-dependent manner through the Nox4-dependent generation of ROS, then influenced the homeostasis of stem cell [89]. When experiencing hypoxia, hyperglycemia could not affect AD-MSC viability and proliferation in AD-MSCs from T2DM patients compared to non-diabetic donors, while keratinocyte growth factor (KGF) which participated in diabetic wound healing was reduced in AD-MSCs from T2DM [90]. A different approach displayed that proliferation activity was enhanced in AD-MSCs from patients with coronary artery disease (CAD) and T2DM; however, the angiogenic activity was significantly declined in these patients which might due to the imbalance of pro- and anti-angiogenic growth factors secreted by AD-MSCs of patients [91]. Interestingly, basic fibroblast growth factor (bFGF) was confirmed to reverse the effect of diabetes on AD-MSCs by improving morphology, increasing proliferative activity, and reducing cellular senescence and apoptosis with decreased oxidative stress [92].

### Reduction of immunosuppression

Metabolic phenotype also influenced the immunomodulatory properties of AD-MSCs. Compared to lean-derived AD-MSCs, obese- and T2D-derived AD-MSCs showed increased expression and secretion of inflammatory cytokines (IL-1b, IL-6, tumor necrosis factor (TNF)-a, and monocyte chemoattractant protein (MCP)-1), activation of the NLRP3 inflammasome, superior migratory, invasion, and phagocytosis capacities, but less effective in suppressing T cell and B cell proliferation, activating the M2 macrophage phenotype, and increasing TGF-b1 secretion [93]. Liu et al. found AD-MSCs from a T2DM ApoE−/− mouse model which performed a hypofunction in suppressing CD4+T lymphocyte proliferation and pro-inflammatory polarization partly due to immune phenotypic changes, cause by AD-MSC immune phenotypic changes with higher MHC-II, CD40, and CD80 [94]. AD-MSCs from obese individuals and mice models can deviate the Th1 response towards the Th17 pathway, increase monocyte-mediated IL-1 secretion, and ultimately lead to impair insulin-mediated Akt phosphorylation and lipolysis inhibition [95].

### Therapeutic effect of diabetic AD-MSCs in vivo

Further mouse model discovered that T2DM mice receiving an intravenous injection of C57BL/6., T2DM, or db/db AD-MSCs improved insulin sensitivity and caused less β cell death. However, db/db AD-MSCs had less effect on increasing insulin sensitivity than C57BL/6and T2DM AD-MSCs. Both T2DM and db/db AD-MSCs had less β cell mass increase, even T2DM AD-MSCs could not improve liver steatosis [15]. What is more serious is that autologous AD-MSCs derived from T2DM patient for the treatment of critical limb ischemia may display peripheral microthrombosis. In vitro study further found diabetic-derived AD-MSCs altered PAI-1 expression levels to blunt fibrinolytic activity. Culture conditions after isolation could not reverse the change, indicating a long-term fibrinolytic modification in T2DM patients [96]. Thus, these studies proved that T2DM might impair the efficiency of autologous stem cell therapy, and force us to find potential approaches to improve stem cell dysfunction.

### Potential approaches to improve MSC function

The microenvironment around stem cells plays a major role in the stem cell function [97]. The regulators in microenvironment mainly included extracellular matrix (ECM), growth factors, and immune cells. The ECM can significantly increase the adhesion and proliferation ability of MSCs. Subsequently, three-dimensional (3D) decellularized ECM culture system was established, which could initiate and sustain the function of stem cells [98]. On the basis of 3D-ECM culture, Block et al. indicated that it is feasible to obtain youthful MSC phenotype in elderly crowd with some markers (e.g., cell size and stage-specific embryonic antigen-4 (SSEA-4) level) [99]. This finding gave us confidence in sorting “rejuvenated” MSCs from age-related disease population, thus improving the therapeutic effect with these high-quality autologous MSCs. Besides, genetically modified MSCs may improve MSC therapeutic potential, have been used in different diseases, including bone diseases, cardiovascular diseases, autoimmune diseases, central nervous system disorders, and cancer [100]. However, clinical trials to evaluate the safety and efficacy of genetically modified MSCs are lacking. To make the potential approaches useful in clinical therapy, more basic and clinical researches are needed.

## Conclusion

Several clinical studies have demonstrated that MSCs may be promising as a new strategy for T2DM. Although the safety and efficacy of MSCs in the treatment of diabetes have been demonstrated in animal studies and some phase I/II clinical trials, there are still many problems to be solved in clinical application. The choice of MSC sources is the fundamental of MSC clinical therapy. As MSCs isolated from different sources all have certain effect on improving T2DM, it is a need to pay more consideration to easy obtainment and no ethical conflicts. These make autologous AD-MSCs an ideal candidate for cell-based therapy. AD-MSCs are capable of differentiating into IPCs, could restore islet β cells, improve insulin resistance, regulate hepatic glucose metabolism, and promote immunosuppression. While further long-term human studies are required to achieve clinical transformation, AD-MSC capabilities can be impaired by hyperglycemia, hyperinsulinemia, and metabolic disturbance which lead to weak auto-transplantation effect and even induce complications. Existing investigations found that pretreatment of MSCs during the in vitro amplification culture phase may alleviate the dysfunction of MSCs isolated from patients. Besides, the selection of appropriate patients also needs attention, as the microenvironment characterized by chronic inflammation in T2DM patients will affect the transplanted cells. In addition, the ideal transplant route was not definitely identified. Systemic infusion may be more effective than targeted therapy for therapeutic effect mainly derived from their secretion function, not from the little homing and differentiation of MSCs in local. Peripheral intravenous injections are easier to operate and have fewer side effects than the targeted approach, especially in multiple injections schedule.

Thus, although several studies supported the potential therapy effect of AD-MSCs in T2DM, large-scale and controlled studies are required to confirm the efficacy and optimal therapeutic scheme before MSC transplantation becomes conventional therapy.

## Data Availability

Not applicable

## Abbreviations

2 h-PBG: 2-h postprandial blood glucose
AD-MSCs: Adipose-derived MSCs
ASCs: Adult stem cells
bFGF: Basic fibroblast growth factor
BM-MNCs: Bone marrow-derived mononuclear cells
BM-MSCs: Bone marrow-derived MSCs
CAD: Coronary artery disease
CB-SCs: Cord blood-derived multipotent stem cells
CITR: Collaborative Islet Transplant Registry
CM: Conditioned media
ESCs: Embryonic stem cells
FPG: Fasting blood glucose
HOT: Hyperbaric oxygen treatment
IPCs: Insulin-producing cells
iPSCs: Induced pluripotent stem cells
IPTR: International Pancreas Transplant Registry
IR: Insulin resistance
KGF: Keratinocyte growth factor
MCP-1: Monocyte chemoattractant protein-1
MSCs: Mesenchymal stem cells
PD-MSCs: Placenta-derived MSCs
ROS: Reactive oxygen species
SAT: Subcutaneous adipose tissue
T2DM: Type 2 diabetes mellitus;
TNF-a: Tumor necrosis factor-a
Treg: Regulatory T
WJ-MSCs: Wharton’s jelly-derived MSCs
